# Laboratory and clinical aspects of human papillomavirus testing

**DOI:** 10.3109/10408363.2012.707174

**Published:** 2012-08-23

**Authors:** Paul K.S. Chan, María Alejandra Picconi, Tak Hong Cheung, Lucia Giovannelli, Jong Sup Park

**Affiliations:** 1 Department of Microbiology, Faculty of Medicine, The Chinese University of Hong Kong, Prince of Wales Hospital, Shatin, New Territories, Hong Kong Special Administrative Region, People's Republic of China; 2 Servicio Virus Oncogénicos, Departamento Virología, Instituto Nacional de Enfermedades Infecciosas, ANLIS “Carlos G. Malbrán”, Buenos Aires, Argentina; 3 Department of Obstetrics and Gynaecology, Prince of Wales Hospital, Hong Kong Special Administrative Region, People's Republic of China; 4 Sezione di Microbiologia, Dipartimento di Scienze per la Promozione della Salute, Azienda Ospedaliera Universitaria Policlinico P. Giaccone, Palermo, Italy; 5 Department of Obstetrics and Gynecology, Seoul St. Mary's Hospital, The Catholic University of Korea, Seoul, Korea

**Keywords:** Human papillomavirus, warts, epidermodysplasia verruciformis, skin cancer, cervical cancer, vaginal cancer, vulvar cancer, oropharyngeal cancer, penile cancer, cervical intraepithelial neoplasia, cervical screening, triage, biomarker, co-test, colposcopy

## Abstract

Human papillomavirus (HPV) infection is associated with a wide spectrum of disease that ranges from self-limited skin warts to life-threatening cancers. Since HPV plays a necessary etiological role in cervical cancer, it is logical to use HPV as a marker for early detection of cervical cancer and precancer. Recent advances in technology enable the development of high-throughput HPV assays of different formats, including DNA-based, mRNA-based, high-risk group-specific and type-specific methods. The ultimate goal of these assays is to improve the accuracy and cost-effiectiveness of cervical screening programs. HPV testing has several potential advantages compared to cytology-based screening. However, since the cancer to transient infection ratio is always low in the general population, HPV test results are bound to have a low positive predictive value that may subject women to unnecessary follow-up investigations. The wide-spread administration of prophylactic HPV vaccine will substantially decrease the incidence of cancer and precancer. This poses a number of challenges to cytology-based screening, and the role of HPV testing is expected to increase. Finally, apart from technical and cost-effiectiveness considerations, one should also keep in mind the psycho-social impact of using sexually-transmitted agents as a marker for cancer screening.

## Introduction

To date, more than 120 types of human papillomavirus (HPV) have been characterized^1^. HPV exclusively infects epithelial cells and is associated with a broad spectrum of clinical manifestations that range from self-limiting lesions to life-threatening disease^2^–^13^.

With the advances in molecular techniques over the last decade, our understanding of this family of ubiquitous viruses has improved tremendously, a number of previously unrecognized important disease associations have been confirmed, and detection assays for epidemiological study and for confirming diagnosis to assist patient management have been developed. Vaccines targeting two HPV types that show the strongest association with cervical cancer have been developed^14^. Assessment of vaccine cost-effectiveness and priority becomes an important task for public health policy makers. To this end, HPV detection and typing assays are indispensable in defining the fraction of disease attributed to the types covered by current vaccines.

## Disease spectrum

### Benign lesions

#### Non-genital skin infections

With the improvement in the sensitivity of detection assays and coverage for a variety of HPV types, it is now recognized that asymptomatic carriage of HPV on healthy skin is common and can persist over several years^15^. Persistence of HPV infection on non-genital skin does not seem to be related to age, sex, immunosuppressive treatment or history of warts^16^,^17^. Clinical manifestations of these lesions are characteristic. In most situations, diagnosis can be made clinically without HPV testing^18^–^19^. Testing for HPV in these lesions is mainly for epidemiological study or other research purposes.

HPV causes benign warts on the skin, which present as flat or firm non-itchy papules^10^. Commonly affected areas include hands (verrucae palmares) and feet (verrucae plantares), which are associated typically with HPV 1, 2, and 4. Skin warts, except those growing over press areas, are non-painful. Verrucae planae are flat skin-colour warts found on the face, hands and forearms, and are caused most commonly by HPV 3 and 7. Periungal warts found at the nail fold are often painful. Butcher's warts are found rarely on the hands of butchers who have repeated trauma that predisposes them to infection with, most commonly, HPV 7; although it is a well-known occupational disease, there is no evidence that the source of infection is animal papillomaviruses^20^.

#### Genitial infections

Genitial tract HPV infection is the most common sexually-transmitted infection. Infections are often subclinical. Clinical lesions (condylomata acuminata) are often multiple and appear as exophytic papillomas, flesh or brown in colour. HPV 6 and 11 are the types most commonly found in visible anogenital warts, but other types, including those typically found in non-genital skin, can also be detected, especially from anogenital areas without visible lesions^3^,^21^–^25^.

#### Oral infections

The oral mucosa is also susceptible to HPV infection. Such infection involving the larynx represents a rare but severe disease^3^,^7^,^26^–^28^. Laryngeal papillomatosis has two age-related incidence peaks; those with early childhood onset are acquired vertically from maternal condyloma, whereas the adult onset group is presumably acquired via orogenital contact. In both groups, HPV 6 and 11 are the most commonly found HPV types. The lesions, which develop mainly over vocal cords and trachea, present as hoarseness of voice and stridor. Lesions may extend to lungs, nose and oral cavity. Treatment is difficult as the lesions often recur; thus it is also known as recurrent respiratory papillomatosis. Heck's disease, another rare condition, presents as multiple papillomas over the lip and buccal mucosa^29^–^31^.

### Malignant lesions

The hypothesis that HPV plays a role in the development of cervical cancer was proposed in the mid-1970s. Over the last 40 years, a strong body of evidence has accumulated to prove the etiological association between HPV infection and a number of human cancers in addition to cervical cancer^11^,^32^,^33^.

#### Epidermodysplasia verruciformis

Epidermodysplasia verruciformis (EV), a genetically-inherited chronic skin condition, presents as disseminated flat warts. Recent studies have shown that some EV patients have mutations in the *EVER1* and *EVER2* genes^34^–^36^. Certain HPV types belonging to the beta genus (HPV 5, 8, 9, 12, 14, 15, 17, 19, 25, 36, 38, 47 and 50) are specifically linked to EV. These EV-associated HPV types are also commonly found in the general population. Some immunosuppressed individuals who do have EV may develop lesions caused by EV-associated HPV types. There is no evidence that EV patients are more susceptible to infection or disease manifestations caused by the alpha genus of HPV. About half of the EV patients develop squamous cell carcinoma in sun-exposed areas. Most malignant lesions are associated with HPV 5 and 8, though the pathogenic mechanism of these HPV types is still not yet clear^8^,^37^,^38^.

#### Non-melanoma skin cancers

In recent years, some evidence suggests that non-melanoma skin cancers including basal cell and squamous cell carcinoma may be linked to cutaneous HPV infection. The suspicion of a viral cause for cutaneous squamous cell carcinoma is based on the observation that its incidence increases dramatically in solid organ transplant recipients receiving long-term immunosuppressive therapy. While sun-exposure is a recognized risk factor, infection with cutaneous HPV (mainly the beta genus) seems to play a role in the development of non-melanoma skin cancers, especially squamous cell carcinoma. HPV types belonging either to beta species 1 or 2 have been detected from squamous cell carcinoma specimens, but unlike anogenital cancers, no predominant HPV types can be identified^39^,^40^. The etiological association between non-melanoma skin cancers and HPV infection is difficult to prove as the same spectrum of HPV types is prevalent among healthy subjects^9^,^41^. Furthermore, HPV may not be required in maintaining the cancer phenotype and it may therefore escape detection in tumour specimens^42^.

The mucosal group of HPV is associated with several malignant conditions. Bowenoid papulosis presents as multiple small flat pigmented papules on the external genitalia. Histologically, it is a *carcinoma-in-situ*, it can evolve to invasive carcinoma, and it is often associated with HPV 16. A subset of intraepithelial neoplasia and carcinoma of the penis, vulva and vagina is also associated with HPV infection, mainly HPV 16^43^.

#### Cancer of vagina

This uncommon cancer in women has an age-standardized rate of 0.3–0.7 per 100,000 worldwide^44^. The limited information about the role of HPV infection and occurrence of vaginal cancer is mostly based on analysis of a few HPV types using fixed tissues^45^. Epidemiological studies indicate that vaginal cancer resembles cervical cancer, and HPV DNA is detected in a majority of vaginal tumours and their precursor lesions. HPV is detected in 82–100% of vaginal intraepithelial neoplasia grade III, and 64–91% of vaginal cancers; as in cervical cancer, HPV16 is the most prevalent type found^45^,^46^.

#### Cancer of vulva

The age-standardized incidence rates of vulvar cancer lie between 0.5 and 1.5 per 100,000. The geographical pattern of vulvar cancer is different from cervical cancer and high rates are observed in several European populations (Scotland, Denmark, Spain, Italy), whereas the prevalence in sub-Saharan Africa, Southeast Asia, and Latin America is low. Distinct subtypes, such as the warty and basaloid types, have been recognized, but the majority of tumours are squamous cell carcinoma. Etiologically, because vulvar carcinomas are heterogeneous, the prevalence of HPV infection in invasive vulvar cancer cases varies^47^. Vulvar cancer with basaloid histopathology in young women is often associated with HPV. HPV 16, 31 and 33 are the most frequently-detected types in this type of vulvar cancer and its precursor lesions^48^. On the other hand, vulvar cancer with verrucus subtype and some cases of precancerous lesions of vulvar intraepithelial neoplasia are not associated with HPV infection^49^. In general, the HPV-positive and -negative groups of vulvar squamous cell carcinoma share a similar prognosis^50^.

#### Cancer of anus

The vast majority of anal cancers is associated with HPV infection. Cancers arising in the anal canal and tumors of the external skin (anal margin) are classified as skin cancers. The canal is lined in its upper part by colorectal-type mucosa, and in its lower third by squamous epithelium, with a specialized transitional zone in between. Therefore, cancers are predominantly squamous cell carcinoma, adenocarcinoma, or basaloid and cloacogenic carcinoma. In most populations, squamous cell carcinoma is twice as common in females as males. However the incidence is particularly high among men who have sex with men and the risk is increased further by infection with human immunodeficiency virus, cigarette smoking, anal intercourse, and more lifetime sexual partners^51^–^53^.

#### Cancer of penis

Globally, this rare cancer accounts for less than 0.5% of all cancers in men. The concordance of cervical and penile cancer in married couples and the geographical distribution of these cancers suggest that they share a common etiology^54^. Serological studies have confirmed the role of HPV 16 and HPV 18 in the etiology of penile cancer, and HPV DNA is detected in 40–50% of such cancers^55^–^57^.

#### Cervical cancer

Among the cancers for which a confirmed or probable etiological link with HPV infection has been established, cervical cancer has the strongest association and accounts for the largest share of disease burden^2^,^12^,^58^–^60^. In 2008, there were about 530,000 new cases of cervical cancer, and about half this number (275,000) died of the disease worldwide; the age-standardized incidences of new cases and mortality were 15 and 8 per 100,000, respectively^61^,^62^. Worldwide, cervical cancer ranked third among cancers in women, just following breast cancer (1.3 million new cases) and colorectal cancer (0.57 million new cases) in 2008. The incidence of cervical cancer varies widely and the developing world accounts for more than 85% of both incidence and mortality. The annual age-standardized incidence rates range from 56 per 100,000 (Guinea) to < 1 per 100,000, depending mainly on the availability of organized cervical screening programs. Overall, the lowest disease burden is recorded from Australia, New Zealand, North America and Western Europe, whereas highest burden is seen in Africa, South-Central Asia and South America^62^.

#### Oropharyngeal cancer

An increase in incidence of oropharyngeal squamous cell carcinoma, specifically those originated from the tonsil and tongue base, has been observed in some parts of the world^63^,^64^. A proportion of these lesions are associated with HPV infection^65^–^68^. The prevalence of HPV in these tumours varies geographically and reflects the variation in prevalence of oral HPV infection, which in turn mainly depends on the practice of oral sex. HPV-positive oropharnygeal squamous cell carcinomas differs from HPV-negative ones in several molecular aspects, reflecting that they are distinct entities. The molecular features observed in HPV-positive oropharyngeal cancers are consistent with the notion that HPV plays a role in the development of these tumours^5^,^6^,^65^,^69^.

## Clinically important basic virology

### Viral genome and key proteins

**Figure 1:**
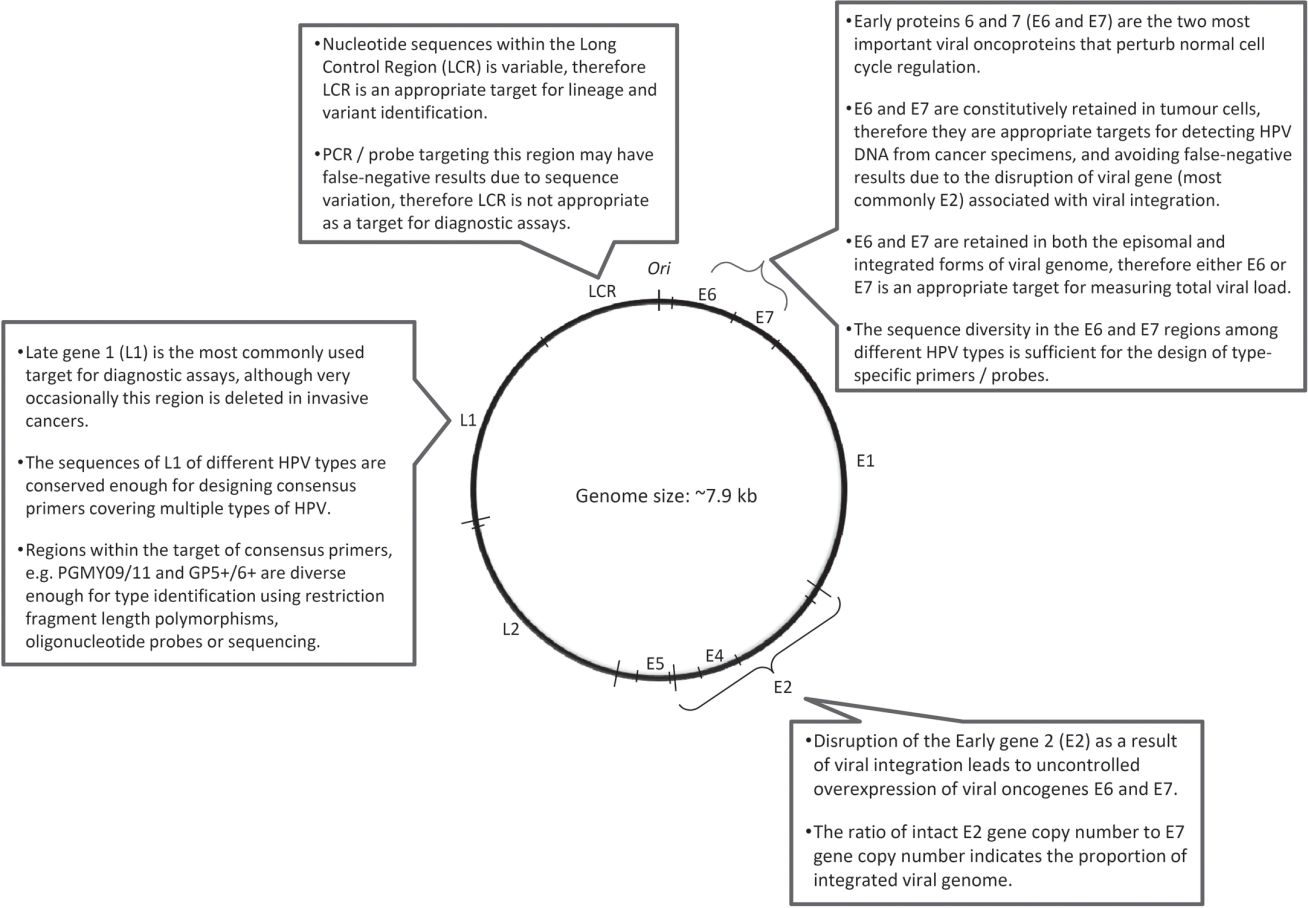
Key targets of human papillomavirus genome for detection.

Papillomaviruses have a small double-stranded DNA genome of about 8 kb in length. The key functions and proteins encoded by different regions of the HPV genome that are relevant for designing detection assays are shown in Figure 1.^70^–^72^ The viral genome has eight open reading frames (ORF) encoding two structural (late) proteins L1 and L2; these form the viral capsid that is about 55 nm in diameter and protects the viral genome inside. L1 is the major structural protein which is conserved within a given HPV type. The fact that L1 protein is immunogenic and conserved within a given type makes it a prime target as antigens for serological assay as well as prophylactic vaccine development^73^. L1 proteins can reassemble themselves under appropriate *in vitro* conditions to form virus-like particles which are the main constituent of current prophylactic vaccines^14^,^53^,^74^–^77^. L2 is the minor capsid protein which can potentially elicit a broader spectrum of neutralizing antibodies against different types of HPV. The potential of using L2 protein as an additional component in future vaccines is being investigated^78^–^81^. The early proteins E5, E6 and E7 encoded by HPV contribute to tumour progression. The oncogenic activities of E6 and E7 are well-characterized. Both E6 and E7 have numerous cellular targets. E6 proteins encoded by high-risk HPV types primarily bind to the tumour suppressor protein p53, and the binding is mediated by the E6-associated proteins (E6-AP). Overexpression of E6, together with its interactions with other cellular proteins, results in the degradation of p53, anti-apoptosis, chromosomal destabilization, enhancement of foreign DNA integration and activation of telomerase^82^–^89^. The E7 proteins encoded by high-risk HPV types also demonstrate an important role in tumourigenesis. E7 binds to a large number of cellular proteins, most importantly the retinoblastoma protein (Rb) and the Rb-related pocket proteins. Such binding results in inactivation of Rb-related pocket proteins, activation of cyclins, inhibition of cyclin-dependent kinase inhibitors, and enhancement of foreign DNA integration and mutagenesis^90^–^95^. The expression of E6 and E7 is tightly controlled via a promoter located at the non-coding Long Control Region (LCR) of the viral genome. Other early proteins encoded by papillomaviruses are E1, E2 and E5. In contrast to E6 and E7, the oncogenic activities of E5 are much less well-defined. Recent studies indicate that E5 plays a role in escape from immune surveillance, upregulation of transcription factors and inhibition of apoptosis^96^–^98^. E2 is another important region of the papillomavirus genome^99^. E2 proteins form complexes with E1 to initiate viral replication. E2 also regulates the expression of E6 and E7, and can exert suppressive or activating effects depending on the abundance of E2. Disruption of E2 ORF as a result of integration of viral genome into the host genome allows an uncontrolled overexpression of viral oncoproteins E6 and E7, which is a hallmark in cervical cancer^100^–^102^.

### Concept of HPV type

HPV are classified under the family *Papillomaviridae* which comprises members infecting humans, non-human primates, birds, and reptiles^1^,^103^–^105^. Unlike many other viruses, the concept of “strain” is not used for papil-lomaviruses as they cannot be grown by conventional cell culture methods. “Serotype” cannot be applied because of the lack of a robust antibody response following natural infection. Thus, HPV types refer to “genotypes”, which are classified based on sequence similarity. To date, more than 120 HPV types have been well-characterized^1^. The L1 ORF is a key region for classification of HPV types (Figure 1). Technically-speaking, the DNA sequence of the L1 region of an HPV type differs from all other types by more than 10%. When the differences in L1 ORF nucleotide sequences between two isolates are within 2–5%, they are regarded as subtypes; differences below 2% are referred as variants. The findings that sequence divergence of a given HPV type is limited and subtypes are rare indicate that HPV has probably gone through genetic drift that became amplified by founder effects and bottlenecks of evolution^106^–^111^. While the classification of HPV “types” is based on genome sequence, it has strong clinical implications^112^,^113^. Firstly, the phylogenetic grouping of HPV types reflects tissue tropism observed in clinical infections. HPV types belonging to the alpha genus (also known as super-group A) mainly infect the mucosal areas, whereas types in the beta genus (also known as supergroup B) mainly infect the cutaneous areas. Secondly, the protective immune response elicited by HPV infection is mainly type-specific. Similarly, the protection induced by the currently available prophylactic vaccines (Gardasil® quadrivalent with HPV 6, 11, 16, 18 from Merck & Co.; Cervarix® bivalent with HPV 16, 18 from GlaxoSmithKline Biologicals) is also mainly type-specific, although some degree of cross-protection, especially for the bivalent vaccine, has been observed^114^–^118^. The ability of HPV to induce cancer is also type-related. HPV types can be grouped into high-risk and low-risk based on their association with the development of cervical cancer. The risk associations identified by epidemiological and biochemical studies are similar. There is no dispute in grouping the commonly-found HPV types, but classification of rare types is still not clear^119^. In view of the current epidemiological, biochemical and phylogenetic data, the mucosal/anogenital HPV types can be grouped as “high-risk” (HPV 16, 18, 31, 33, 35, 39, 45, 51, 52, 56, 58 and 59), “probable high-risk” (HPV 68), “possible high-risk” (HPV 26 and 73), and “low- or unknown risk” (HPV 6, 11, 30, 34, 40, 42, 43, 44, 53, 54, 55, 57, 61, 62, 66, 67, 69, 70, 82, 85 and 97)^2^,^105^,^112^,^119^,^120^.

## Virus detection approaches

Because the replication cycle of papillomaviruses can be completed only in differentiated epithelial cells, isolation of viruses from clinical samples is difficult^72^. Growth of papillomaviruses has been accomplished only in a specialized primary human keratinocyte-derived cell culture system, organotypic “raft” culture, where epithelial cells are grown on semi-solid agar to allow differentiation of epithelial cells and hence productive replication of papillomaviruses^121^–^123^. Even with this technique, only a few types (mainly HPV 11 and 31) have been replicated successfully in laboratory conditions.

HPV is equipped with several immune evasion mechanisms. It multiplies in keratinocytes that have a short-life span, and thus the progeny viruses can be released in a natural way without inducing cell lysis that is seen with other non-enveloped viruses. Tis avoids triggering of infammation and immune responses associated with cellular damage. The lack of a viremic phase also minimizes stimulation to the systemic immune system. Furthermore, the virus actively downregulates the synthesis of interferon. As a result, although the immune system plays an important role in clearing infection and it is associated with a strong localized cellular immune response, natural infection with papillomaviruses does not result in a robust antibody response^124^–^130^. For this reason, serological diagnosis has limited clinical and epidemiological value. For instance, only about 50–70% of women with persistent cervical HPV infection mount a detectable antibody response, and most women with transient infection do not have detectable antibodies or have them only for a short time^131^–^135^. Thus, the detection of HPV infection relies mainly on a molecular approach that amplifies the viral genome or mRNA, or detection of viral protein using immunoassays.

### DNA-based assays

Because HPV cannot be cultured, all HPV assays currently in use rely on the detection of viral nucleic acids. They can be divided into: 1) target-amplification methods (PCR [polymerace chain reaction] with consensus or type-specific primers, and HPV mRNA amplification), and 2) signal amplification methods (liquid-phase or *in situ* hybridization). It is necessary to distinguish analytical sensitivity (minimum number of HPV genomes to be present in a sample to generate a positive test result) from clinical sensitivity (proportion of women with disease who test positive) (Table 1).

**Table 1:** Parameters for assessing the clinical performance of HPV tests in cervical cancer screening.

Terminology	Characteristics to measure
Sensitivity^1^	The proportion (expressed as percent) of patients ***with*** a certain disease (e.g. CIN III or invasive cervical cancer) who have a POSITIVE HPV test. E.g., a sensitivity of 95% for CIN III means that 5 of 100 CIN III cases will be NEGATIVE (false-negative) and will be missed by this test. Sensitivity is affected mainly by the test itself and to some extent by the nature of samples submitted for testing.
Specificity^2^	The proportion of subjects (expressed as percent) ***without*** a certain disease (e.g. CIN II or higher severity) who have a NEGATIVE HPV test. E.g., a specificity of 70% means that if 100 women without CIN II or higher severity participate in the screening, 30 will be POSITIVE (false-positive) by the test. Specificity is affected mainly by the test itself and to some extent by the nature of samples submitted for testing.
Positive predictive value	The chance (expressed as percent) that a ***POSITIVE HPV*** test result indicates the presence of a certain disease (e.g. invasive cervical cancer). In the context of cervical screening, this predictive value can be further divided into predictive value for the current situation or in the next 5 or 10 years. The positive predictive value of a test depends mainly on the prevalence of the disease in the target population. Cancer screening tests, including HPV, are bound to have low positive predictive value when applied to the general population.
Negative predictive value	The chance (expressed as percent) that a ***NEGATIVE HPV*** test result excludes the presence of a certain disease (e.g. CIN II or higher severity). Under the context of cervical screening, this predictive value can be further divided into predictive value for current situation or in the next 5 or 10 years. The negative predictive value of a test depends mainly on the prevalence of the disease in the target population.

1 The definition presented here refers to “clinical” sensitivity. From the laboratory performance point of view, the concept of analytical sensitivity is often used, which means the lowest amount of analyte required in the specimen to generate a positive result, (also referred to as detection limit, e.g. 100 copies of viral DNA). For the HPV test, increasing the analytical sensitivity may not always increase the clinical sensitivity, but usually specificity is lost.

2 From the laboratory performance point of view, “specificity” means cross-reaction with similar targets. This is more relevant for assays intended for HPV typing. e.g. an assay with poor specificity may misidentify an HPV 58–positive sample as HPV 33.

The choice of the HPV test depends on the application (Table 1). Assays with high analytical sensitivity are crucial for molecular epidemiological studies and for evaluating vaccine efficacy. HPV typing assays with high analytical sensitivity and specificity are the key in virological surveillance, including the evaluation of vaccination impact on the prevalence of vaccine-covered types, identification of new types, discrimination of types in multiple infection, and monitoring of potential type replacement in the post-vaccine era.

On the other hand, when applied in the clinical situation for cervical cancer screening and post-treatment follow-up, assays with lower analytical sensitivity may produce a better positive predictive value^136^–^138^. It is important that HPV tests are clinically validated under context-specific conditions, especially in the target population.

### Hybrid capture HPV DNA assay

Hybrid capture (HC2) is based on liquid phase hybridization using long synthetic RNA probes complementary to the genomic sequences of 13 high-risk types (HPV 16, 18, 31, 33, 35, 39, 45, 51, 52, 56, 58, 59 and 68) and five low-risk types (6, 11, 42, 43 and 44); these are used to prepare high-risk (B) and low-risk (A) probe cocktails that are used in two separate reactions^139^. In practice, often only the high-risk probes are used. DNA present in the biological specimen is hybridized in solution phase with each of the probe cocktails and forms specific HPV DNA-RNA hybrids. These hybrids are then captured by antibodies bound to the wells of microtiter plates that recognize specific RNA-DNA hybrids. After removal of excess antibodies and non-hybridized probes, the immobilized hybrids are detected by a series of reactions that give rise to a luminescent product that is detected by a luminometer. The intensity of emitted light, expressed as relative light units, is proportional to the amount of target DNA present in the specimen, and provides a semiquantitative measure of the viral load. The HC2 is currently available in a 96-well microplate format with automation that is easy to use in clinical settings. An automated robotic platform that allows handling of 96-well microplates has been developed for high-volume testing.

HC2 does not require special facilities to avoid cross-contamination because it does not rely on target amplification to achieve high sensitivity, as do PCR protocols. The recommended cut-of value for test-positive results is 1.0 relative light unit (equivalent to 1 pg HPV DNA per 1 mL of sampling buffer). Several studies have noted that the high-risk probe cocktail in HC2 cross-reacts with HPV types that are not represented in the probe mix. It has been reported that HC2 using the high-risk probe at a 1.0-pg/mL cut-off detected HPV types 53, 66, 67, 73, as well as other undefined types, and raising the cut-of to 10.0 pg/mL did not eliminate the cross reactivity to types 53 and 67^140^. Cross-reactivity of HC2 high-risk probe to HPV types that have a signifcant risk for cervical cancer may be considered as beneficial, but cross-reaction with low-risk types causing false-positive results may decrease the specificity and positive predictive value^141^.

#### Consensus, group- or type-specific PCR

**Figure 2:**
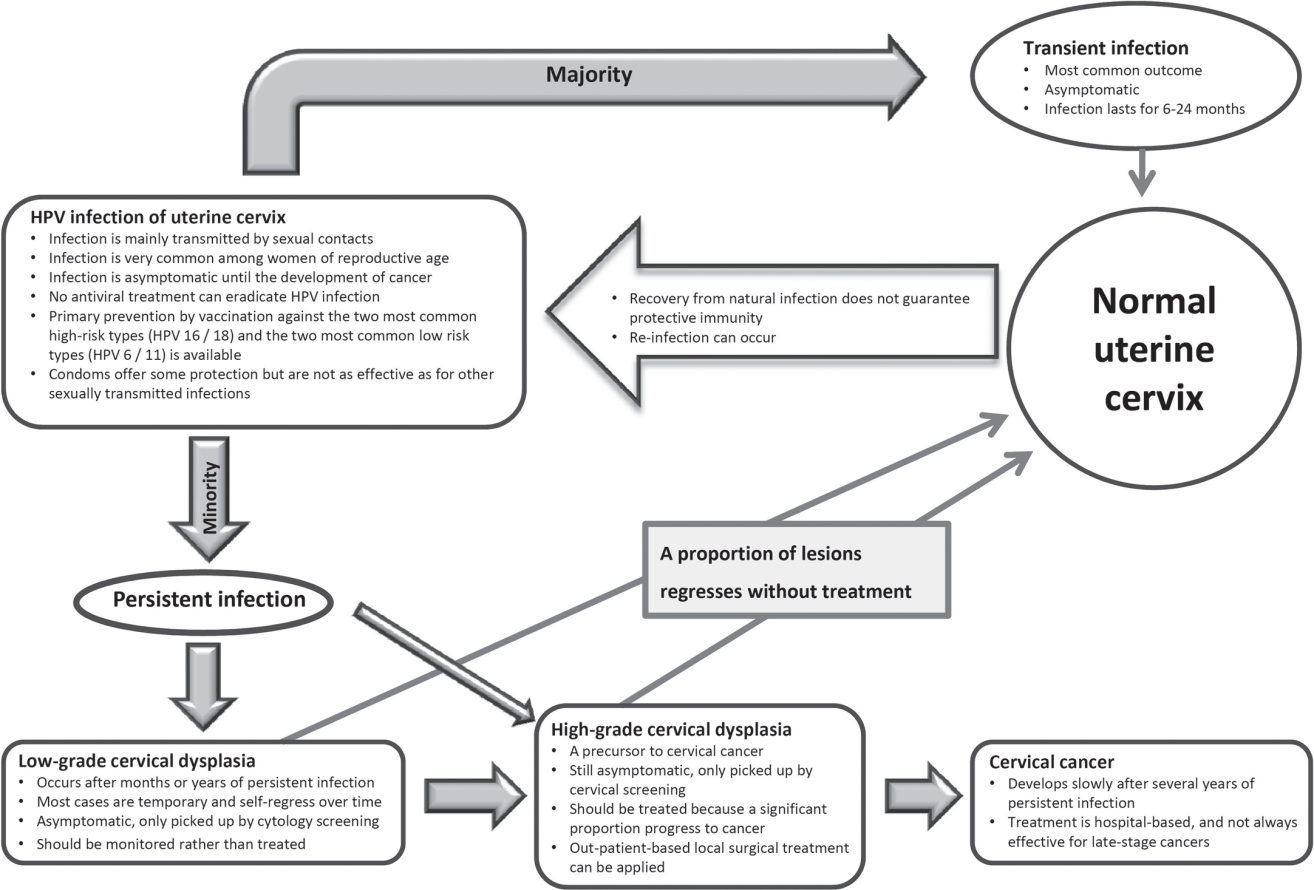
Natural history and management of cervical HPV infection.

Currently, the most important clinical application of HPV detection is to identify women with a higher risk of developing cervical intraepithelial neoplasia or invasive cervical cancer (Figure 2). HPV DNA detection systems that are designed to catch all the mucosal or anogenital HPV types are referred as “consensus”, those detecting high-risk or low-risk HPV types as a group are referred as “group-specific”, and those identify individual HPV types are referred as “type-specific”. A variety of commercial assays belonging to each category are available (Table 2).

**Table 2:** Commercially a vailable HPV tests.

Molecular target	Type differentiation	Test name	Principle	Manufacturer
HPV DNA (full genome)	High-risk types as a group, not type-differentiating	Hybrid Capture 2 HPV DNA Test	Hybridization	QIAGEN, Gaithersburg
		CareHPV Test	hybridization	QIAGEN, Gaithersburg
HPV DNA (L1 ORF)	High-risk types as a group, not type-differentiating	Amplicor HPV Test	PCR	Roche, Branchburg
		Cervista HPV HR Test^1^	Hybridization (Invader)^2^	Hologic, Madison
HPV DNA (L1 ORF)	Differentiate 13 or more high-risk types	CLART	Reverse line-blot Hybridization on PCR products	Genomica, Coslada
		INNO-LiPa HPV Genotyping	Reverse line-blot Hybridization on PCR products	Innogenetics, Gent
		Linear Array HPV Genotyping Test	Reverse line-blot Hybridization on PCR products	Roche, Branchburg
		*Digene* HPV Genotyping RH Test (RUO)	Reverse line-blot Hybridization on PCR products	Digene, Hilden
HPV DNA (E1 ORF)	Differentiate 13 or more high-risk types	Infniti HPV-HR QUAD Assay	Microarray on PCR products	Autogenomics, Carlsbad
		PapilloCheck	Microarray on PCR products	Greiner Bio-one, Frickenhausen
HPV DNA (L1 ORF)	Limited type differentiation - HPV 16/18	Cervista HPV Test^1^ CORBAS 4800 HPV Test Real Time High Risk (HR) HPV Test	Hybridization Real-time PCR Real-time PCR	Hologic, Madison Roche, Pleasanton Abbott, Des Plaines
HPV E6/E7 mRNA	14 high-risk types as a group, not type-differentiating	APTIMA HPV Assay	TMA	GenProbe, San Diego
HPV E6/E7 mRNA	Limited type differentiation – HPV 16/18/31/33/45	NucliSENS EasyQ HPV^3^	NASBA	BioMerieux, Marcy-l'Étoile
		PreTect HPV-Proofer^3^	NASBA	Norchip, Klokkarstua

NASBA = nucleic acid sequence-based amplification; TMA = transcription-mediated amplification.

1 Also targets E6 and E7.

2 The “Invader” reaction involves two simultaneous isothermal reactions. A primary reaction is based on hybridization with two sequence-specific oligos to the same target, creating a single-nucleotide overlap. The overlap together with its 5' flap will be cleaved. In the secondary reaction, the cleaved flap combines with a fuorescence resonance energy transfer (FRET) probe that generates a fuorescent signal. As a result, each released 5' flap from the primary reaction cycles on and of the FRET probes, enabling the secondary reaction to further amplify the target-specific signal to 1–10 million-fold.

3 Same technology marketed under different brand names in different countries.

#### Target regions

*L1*: The L1 region is the most frequently-used target for amplifying HPV genome from clinical samples (Figure 1). L1, on the one hand, is conserved enough for the design of consensus primers to amplify a broad spectrum of HPV types by using a single set of degenerated primers or a cocktail of primers^142^–^149^. The commonly-used primer sets are shown in Table 3. On the other hand, the L1 region also has sufficient sequence diversity to allow the identification of individual HPV types based on further analysis of the amplified products. Given the definition that L1 sequences of different HPV types should differ by more than 10%, type differentiation is achievable even based on a short fragment, less than 100-bp, of L1. HPV type can be identified by a few approaches. Type-specific restriction fragment length polymorphisms can be generated by using two restriction endonucleases Rsa I and Dde^150^,^151^. This method was more popular when sequencing was still expensive and labour intensive. The main disadvantage of restriction fragment length polymorphisms is the ambiguous restriction fragment patterns generated from co-infection with multiple types, especially when three or more types are present in a sample. This approach has gradually been replaced by sequencing because the cost for the latter has fallen substantially in recent years. However, sequencing of PCR product can reveal only the HPV type that predominates in a co-infection. Today, the best approach to identify multiple HPV types simultaneously from a sample is to perform hybridization with multiple HPV type-specific probes immobilized on strips, membranes or array slides (Table 2).

**Table 3:** Commonly used primers targeting the L1 region of HPV genome.

Primer name	Primer sequences	Remarks	References
MY09/11	Forward (5'-GCMCAGGGWCATAAYAATGG-3') Reverse (5'-CGTCCMARRGGAWACTGATC-3')	One of the commonly used first generation primers. Target a ∼450-bp fragment which is usually too long for formalin-fixed tissues. The amplified product allows type identification by restriction fragment length polymorphisms, hybridization with type-specific probes or by sequencing.	143
PGMY09/11	PGMY11-A (5'-GCA CAG GGA CAT AAC AAT GG-3') PGMY11-B (5'-GCG CAG GGC CAC AAT AAT GG-3') PGMY11-C (5'-GCA CAG GGA CAT AAT AAT GG-3') PGMY11-D (5'-GCC CAG GGC CAC AAC AAT GG-3') PGMY11-E (5'-GCT CAG GGT TTA AAC AAT GG-3') PGMY09-F (5'-CGT CCC AAA GGA AAC TGA TC-3') PGMY09-G (5'-CGA CCT AAA GGA AAC TGA TC-3') PGMY09-H (5'-CGT CCA AAA GGA AAC TGA TC-3') PGMY09-I (5'-GCC AAG GGG AAA CTG ATC-3') PGMY09-J (5'-CGT CCC AAA GGA TAC TGA TC-3') PGMY09-K (5'-CGT CCA AGG GGA TAC TGA TC-3') PGMY09-L (5'-CGA CCT AAA GGG AAT TGA TC-3') PGMY09-M (5'-CGA CCT AGT GGA AAT TGA TC-3') PGMY09-N (5'-CGA CCA AGG GGA TAT TGA TC-3') PGMY09-P (5'-GCC CAA CGG AAA CTG ATC-3') PGMY09-Q (5'-CGA CCC AAG GGA AAC TGG TC-3') PGMY09-R (5'-CGT CCT AAA GGA AAC TGG TC-3')	An improved version of MY09/MY11 targeting the 144 same 450-bp fragment. Use in the Linear Array HPV Genotyping Test (Roche, Branchburg), where the HPV types are identified by hybridization with type-specific probes.	144
GP5+/6+	Forward (5'-TTT GTT ACT GTG GTA GAT ACT AC-3') Reverse (5'-GAA AAA TAA ACT GTA AAT CAT ATT C-3')	Targets a ∼150-bp fragment suitable for formalin-fixed tissues. Commonly used in large-scale epidemiological studies. Reported to have sub-optimal sensitivity for a variant of HPV 52 commonly found in East Asia due to mutation at the primer binding site.	145,153
SPF	Forward primer (5'-GCI CAG GGI CAT AAC AAT GG-3') Two reverse primers: (5'-GTI GTA TCI ACA ACA GTA ACA AA-3') and (5'-GTI GTA TCI ACA TCA GTA ACA AA-3')	Targets a short (∼65-bp) fragment. Good for samples with fragmented DNA such as formalin-fixed tissues. Modifed versions include SPF6 and SPF10 with the addition of more primers. SPF10 primers are used in the INNO-LiPa HPV Genotyping assay, but sequences not disclosed.	146
L1F/L1R	Forward (5'-CGT AAA CGT TTT CCC TAT TTT TTT-3') Reverse (5'-TAC CCT AAA GAC CCT ATA CTG-3')	Targets a ∼255-bp fragment, a length that may not be retained in formalin-fixed tissues.	147

While L1 is regarded as the best target for HPV genome detection, it is absent from a small proportion of invasive cervical cancer samples, probably due to disruption resulting from viral genome integration^152^. When such a situation is suspected, the constitutively retained genes, E6 or E7, can be amplifed to verify the presence of HPV infection. Furthermore, consensus primer sets may have different analytical sensitivities for different HPV types or variants^147^. When adopting assays developed elsewhere, evaluation based on samples collected from the local population is necessary before the assays are used clinically.

*E6, E7:* The E6 and E7 regions are good alternative targets for amplifying HPV genome from clinical specimens. Firstly, the nucleotide sequence diversity of E6 and E7 between HPV types allows the design of type-specific primers. Secondly, E6 and E7 gene expression is required to maintain the transformed phenotype of infected cells^153^–^159^. Therefore, both E6 and E7 genes are expected to be retained regardless of the status of viral integration. The constitutional presence of E6 and E7 genes makes them an appropriate target for viral load quantification^160^,^161^.

*E2:* Papillomavirus infection can exist in two forms. The vegetative phase, also known as the replicative or productive phase, is associated with a full episomal form of viral genome. This replicative phase is typically found in the upper layers of epithelium with differentiated keratinocytes, or in benign condyloma where a large amount of virus is released^32^,^162^–^164^. In the integrated form, the viral genome is disrupted and therefore the viral replication cycle cannot be completed. It is widely accepted that HPV-mediated cervical carcinogenesis proceeds via the integration of viral genome and disruption of the E2 ORF, thus releasing the suppressive control of E2 on the expression of viral oncogenes E6 and E7. Thus, E2 is often used as a surrogate marker to indicate the status of viral integration. By measuring the ratio between E2 and E6 (or E7) gene copy numbers, one can estimate the proportion of integrated viral genome present in a clinical specimen.

### E6/E7 mRNA-based assays

E6 and E7 are oncoproteins involved in carcinogenesis. Persistent expression of E6 and E7 could serve as an indicator of progression from intraepithelial neoplasia to invasive cancer^165^–^167^. Detecting the mRNA encoded by E6 or E7 may, therefore, provide a better predictive value for malignant or high-grade lesions^168^–^171^. A few commercial assays based on this approach have been developed recently (Table 2)^172^,^173^. The PreTect HPV-Proofer (Norchip, Klokkarstua) and NucliSENS Easy Q HPV (BioMerieux, Marcy-l'Étoile) assays are based on the same technology, and are marketed under different brand names in different countries. PreTect Proofer and NucliSENS Easy Q detect E6/E7 mRNA from five high-risk types (HPV 16, 18, 31, 33 and 45) commonly found in high-grade lesions and cancers. The APTIMA HPV Assay (Gen-Probe) provides a broader coverage and targets mRNA of 14 high-risk HPV types. In a review on 11 studies examining these three currently-available commercial assays, the sensitivities for cervical intraepithelial neoplasia grade II and above (CIN [cervical intraepithelial neoplasia] II+) lesions ranged from 41% to 86% for the PreTect Proofer/EasyQ assay and from 90% to 95% for the APTIMA assay, whereas the specificities ranged from 63% to 97% for the PreTect Proofer/Easy Q assay and from 42% to 61% for the APTIMA assay^174^.

### E6/E7 protein-based assays

Similarly, measuring the E6 or E7 proteins may provide a better predictive value than detecting viral DNA alone^175^,^176^. However, E6 and E7 proteins are known to be produced in small amounts in transformed cells. The sensitivity of the E6/E7 protein-based assay is a concern that needs to be addressed. Data on clinical evaluation of the E6/E7 protein-based assay is not yet available.

### Building quality control capacity

The need to monitor vaccine effectiveness by effective surveillance programs and the increasing use of HPV assays in the field have stimuated the World Health Organization (WHO) to develop a structured Global Laboratory Network (WHO HPV LabNet). To date, this LabNet includes two Global Reference Laboratories (Sweden and USA) and eight Regional Reference Laboratories (Argentine, Australia, India, Japan, South Africa, Switzerland, Tailand and Tunisia). Work is currently being conducted in the areas of scientific and technical advice, quality assurance, training, and communication (http://www.who.int/biologicals/areas/human_papillomavirus/WHO_HPV_LabNet/en/index.html. Accessed on 9 July 2012). LabNet, with the collaboration of the National Institute of Biological Standards and Control (UK), is establishing international standards (IS) for HPV types, to harmonize HPV nucleic acid amplification technology-based assays. These efforts will be immensely helpful in monitoring the impact of HPV vaccination programs worldwide and in evaluating data on uniform platforms. One of the main aims is to harmonize various HPV detection assays and to minimize inter-laboratory variation by collectively establishing strong and effective quality control and quality assurance programs.

## Clinical applications

### HPV for primary screening

HPV infection progresses slowly to cervical cancer through stages of pathological changes that can be recognized from exfoliated cervical cells (Figure 2). Detection of lesions at the precancerous stage allows intervention by office-based local surgery. Until recently, cervical screening has been based solely on cytological examination of exfoliated cervical cells. The benefit of regular cervical cytology screening is undisputed^177^–^181^. Cervical cancer incidence decreases substantially after the introduction of cervical screening and there are consistent and marked differences in cervical cancer incidence rates between countries with and without organized screening programs^61^,^182^. While conventional Pap smear or liquid-based cytology is still the standard of care in many parts of the world, the intrinsic drawbacks of cytology-based screening call for replacement by HPV testing or the addition of adjunct markers^183^. Compared to molecular markers, cytology is more subjective and requires a stringent quality control and quality assurance program to maintain clinical performance. Cytology is relatively insensitive and is associated with an unavoidable portion of non-specific and self-limiting abnormal results. With the increased availability of high-through-put screening platforms (Table 2)^184^, a large number of large-scale studies have been conducted to investigate the value of using HPV DNA detection (mainly based on hybridization (hybrid capture) or PCR amplification targeting the L1 region) as a primary or supplementary tool for cervical screening.

#### Advantages

The overall advantages of HPV testing over cytology for screening of cervical cancer are: feasibility for high throughput, greater objectivity in result interpretation, high sensitivity, high negative predictive value, and ability to provide long-term risk stratification^185^–^192^. Furthermore, the performance of HPV assays is less subject to variation across centers. For instance, the reported sensitivity of cytology ranges from 33.8% to 94.0%, whereas that of the HPV assay (HC 2), used in the same series of studies across different continents, varies from 84.9% to 97.6%^193^–^199^. However, HPV infections are transient in most women and the prevalence of high-grade intraepithelial neoplasia or cancer among infected women is low. The low specificity and low positive predictive value are the major drawbacks of applying HPV DNA testing in clinical practice. A fine balance has to be established between the sensitivity and specificity of the HPV test to achieve a clinically-useful predictive value (Table 1).

#### Approaches to improve positive predictive value

##### Infection and disease prevalence

**Figure 3:**
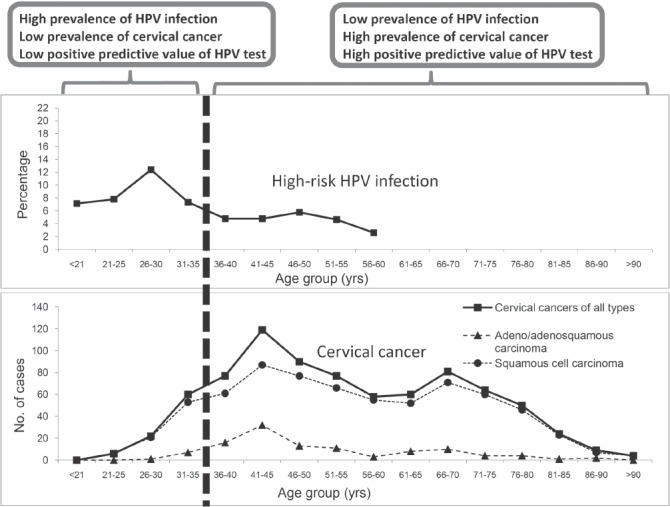
Improved positive predictive value of HPV test by refining the target population. Figure shows the age-specific prevalence of high-risk HPV infection and age-specific distribution of cervical cancer cases in Hong Kong^196^. Restricting HPV test to women aged ≥ 35 years avoids the age-related infection peak, and covers women with higher incidence of cervical cancer. By setting the testing population to women aged ≥ 35 years (in case of Hong Kong as used in this example) will therefore improve the positive predictive value of HPV testing for cervical cancer.

Several approaches can be considered to minimize the “background noise” and to improve the positive predictive value. The “background noise” depends on the prevalence of transient HPV infection among the target population for which the HPV test is being applied. Data on the age-specific prevalence of HPV infection and the age-specific incidence of CIN II/III and cervical cancer are essential to derive a cut-of for applying HPV testing (Figure 3)^200^. In general, HPV testing is not recommended for women below 30 years of age (or 35 in some countries) for whom transient infection is common^187^,^201^,^202^.

##### Super high-risk HPV types

At least 15 HPV types can be linked to cervical cancer with various degrees of risk association^112^. Initially, most HPV assays are designed to detect as many high-risk types as possible. In fact, the risk association among HPV types classified under this so-called “high-risk” group varies substantially. In recent years, it has been recognized that covering the HPV types with a small risk may jeopardize the overall positive predictive value of the assay. Thus type-specific assays, especially those targeting a group of HPV types with the highest risk association, have emerged. In this regard, HPV 16 and HPV 18 should be included as they are the two high-risk types most commonly found in cervical cancers across the world. HPV 31, 33 and 45 are the next group to be included, although their ranking showed some degrees of geographical variation^203^–^207^. HPV 52 and HPV 58 showed an even more skewed geographical distribution, and the clinical value of including these types into screening assays should be assessed based on the HPV type distribution data derived from the target population^152^,^204^,^207^–^215^.

An HPV16/18-specific test is expected to provide the highest positive predictive value. In the most recent guidelines from the United States, HPV16/18 testing is one of the options to triage women found to be HPV-positive but cytology-negative in primary screening using the co-test approach^202^.

##### Persistent infection

Since persistent infection is a pre-requisite for the development of cervical precancer and cancer, one can improve the positive predictive value by considering HPV testing results performed on specimens collected more than 12 months apart. If HPV is detected in both instances, the chance that it is a persistent rather than transient infection is higher. In this regard, an HPV type-specific test is necessary to differentiate repeated infections with different HPV types from genuine persistent infection with the same HPV type(s). It is only the latter that carries an increased risk of cancer development^216^. However, the limitation of this approach is that a proportion of women may not come back for the second test and may become lost to follow-up.

##### Reflex follow-up test

Another approach to improve the positive predictive value of HPV testing as a primary screening tool is to carry out a “reflex” follow-up test for HPV-positive samples. The “reflex” approach can prevent an extra visit and unnecessary anxiety while waiting for the follow-up test results. Biomarkers indicating the transforming activity of HPV can potentially serve this purpose^102^,^217^,^218^. The first approach is to detect direct indicators of HPV oncogene expression. At present, this mainly refers to the detection of mRNA encoded by the viral oncogenes, *E6* and *E7*. Among women with normal cytology, atypical squamous cells of undetermined significance (ASCUS) and low-grade squamous intraepithelial lesions (LSIL), E6/E7 mRNA was detected in 30% of the HPV 16, 56% of the HPV 18 and 75% of the HPV 31 DNA-positive women, respectively. The mRNA test therefore potentially has a higher specificity compared to the HPV DNA test, and thus fewer patients would be referred for further testing or close follow-up^219^. Assays for direct measurement of E6 and E7 proteins from clinical specimens have been described and their clinical performance is being evaluated. The second approach is to detect biomarkers of increased cellular proliferation and chromosomal instability or those upregulated in response to HPV-encoded oncoproteins, including p16^INK4a^, Ki-67, topoisomerase IIA (TOP2), and minichromosome maintenance proteins (MCMP). Among these, p16^INK4a^ is the most promising^220^,^221^. In many non-HPV-associated tumours, p16^INK4a^ is inactivated by genetic deletion or hypermethylation, which leads to an increase in cyclin-dependent kinase activity and inactivation of Rb^222^. In contrast, in HPV-associated tumours, including cervical intraepithelial neoplasia and invasive cervical cancer, the inactivation of Rb by E7 leads to a marked overexpression of p16^INK4a^ as a result of the lost of negative feedback regulation that depends on Rb activity^223^–^225^. It has been shown that, among 425 Pap-negative and HPV-positive women greater than 30 years of age, 25.4% were positive for p16/ki67 dual staining. The dual staining gave a sensitivity of 91.9% for CIN II and 96.4% for CIN III+ after a mean follow-up of 13.8 months (1–27 months)^226^. The result is encouraging but further studies are needed to support its clinical use.

##### Viral genome characterization

The patterns of viral integration at different stages of neoplastic progression have been investigated. Diverse, even conflicting, results have been reported. Some studies observed viral integration mainly from specimens of high-grade lesions^227^,^228^, whereas others found that viral integration takes place early during the course of infection and is detected in a substantial proportion of low-grade lesions^229^–^232^. It has been suggested that viral integration is a consequence rather than a cause of chromosomal instability^233^. Based on the available data, the main concern appears to be the lack of specificity. Basically, viral integration can be detected in normal and low-grade lesions, whereas cervical samples from cases of invasive cancer can harbour purely the episomal form of viral genome^234^–^237^.

DNA methylation is an epigenetic event that is linked to cancer development. A number of studies have been conducted to examine the association between the viral DNA methylation pattern and lesion severity. However, as with viral integration, most of the available data suggests that the patterns observed from low-grade and high-grade lesions are too diverse to achieve a clinically-useful predictive value^238^–^242^. Nevertheless, a recent study using a newer approach, pyrosequencing, has produced some promising results^243^. Viral integration and methylation have a strong biological basis, and further studies to explore their clinical application are worthwhile.

### Co-test with cytology for primary screening

Co-test refers to the use of both HPV and cytology tests in parallel as first-line screening. The main advantage of co-test is the improvement in sensitivity for CIN II+ lesions; women who are double negative will have an extremely low-risk for CIN II+. The potential gain in cost-effectiveness from the expense of the extra test is the longer interval of safety supported by a double negative result. Data have suggested that the safety interval following an HPV-negative result could be as long as 5–7 years^244^–^248^. The most recent guidelines from the United Sates recommend HPV and cytology co-testing every 5 years for women aged 30–65 years^201^. However, the management of women with normal cytology but an HPV-positive result is an issue that is not yet completely resolved. The question is what proportion of the HPV-positive, cytology-normal women have transient HPV infection, and how many of them will turn out to be HPV-negative when the co-test is repeated in 1 year. This is fundamentally the same question when HPV testing is applied alone as a primary screening tool, and the answer varies substantially with the analytical sensitivity of the HPV assay and the prevalence of CIN II/III in the target population^249^. For patients with a normal Pap smear but an HPV-positive test, their risk of developing abnormal cytology and CIN II+ lesions are signifcantly higher than those with a double negative result. The increased risk was found to be HPV type-dependent, and the cumulative risk of those infected with HPV 16 reached 26% after a 13-year follow-up^250^. The United States Food and Drug Administration (US FDA) has approved the use of an HPV16/18 type-specific test in this particular clinical situation for women ≥ 30 years of age in order to identify individuals with a higher risk for developing disease in the future. According to the recent guidelines from the United States, women with HPV-positive and cytology-negative screening results can be either followed with the co-test 12 months later or triaged with the HPV16/18-specific test for referral to colposcopy^201^.

### HPV as a triage for abnormal cytology

The first clinical application of HPV testing was on the triage of patients presented with ASCUS on Pap smear. Patients who were HPV-positive would be referred for colposcopy whilst those who were HPV-negative could be followed by repeating the Pap smear 12 months later^251^–^254^. This is still the most common application of HPV testing, and a large body of evidence is available to support returning women with negative HPV DNA results to a normal screening schedule.

The cost-effectiveness of using HPV testing to triage women with LSIL depends mainly on the context. For instance, it has been shown that about 80% of patients presenting with LSIL were HPV-positive; thus triage of LSIL by HPV testing was not recommended^255^. On the other hand, the US FDA has approved the use of HPV testing in post-menopausal women presenting with LSIL since the prevalence rate of HPV is low in this subset of patients. Therefore, data generated from context-specific studies are very important. The main variable is again the prevalence of HPV infection among the population tested. Such an approach may be effective for women well beyond the peak of infection, so that a substantial proportion of women will have an HPV-negative result and can be reassured^256^,^257^.

The underlying risk of having CIN II+ lesions among women with high-grade squamous intraepithelial lesions (HSIL) or atypical squamous cells is high, but HSIL (ASC-H) cannot be excluded, and colposcopy may be indicated for CIN II+ even if the HPV test result is negative^258^,^259^. HPV testing is also not very useful for women with atypical glandular cells because the underlying pathology may reside in the uterus. Whilst a positive HPV test suggests a cervical lesion, a negative test does not rule out such conditions as endometrial hyperplasia or cancer that are not HPV-related, and the risk of these is substantial in post-menopausal women^260^.

### HPV for post-treatment surveillance

CIN II is often regarded as a threshold for treatment (Figure 2). The predominant mode is excision of the transformation zone using the loop electrosurgical excision procedure (LEEP). Since about 5 to 10% of patients have persistent or recurrent CIN II+ after LEEP, continual surveillance after treatment is needed. When compared to conventional cytology, HPV testing is more sensitive, carries a higher negative predictive value for recurrent or residual lesions, and is recommended by the American Congress of Obstetricians and Gynecologists for post-treatment follow-up^261^–^263^. In a cohort of 917 patients treated by LEEP, 81% were double-negative at 6 months after treatment and their risk of being diagnosed with CIN III+ was low. It has been suggested that patients with both negative cytology and negative HPV DNA can return to the 3-year screening program^264^.

## Future challenges

The currently available prophylactic HPV vaccines are highly effective for the prevention of HPV16/18-related cervical neoplasia and offer some degree of cross-protection for lesions caused by related HPV types. A number of countries have implemented vaccination programs for adolescents and the coverage is expected to increase in the coming years^265^. These countries are also likely to be those running organized cervical screening programs^265^–^268^.

In the coming decades, several issues regarding cervical screening will need to be addressed^269^,^270^. The decrease in absolute incidence of cervical intraepithelial neoplasia and cervical cancer among those vaccinated will jeopardize the positive predictive value of any screening test. To achieve maximum cost-effectiveness, the screening strategy for those who have received the vaccine before their sexual debut should be different from those who have not^271^. These two populations will co-exist for a few decades and it will be a challenge to public health professionals to organize two systems in parallel for the same disease. Among those vaccinated, the chance of detecting a genuine abnormal signal (abnormal cytology results representing cervical precancer or cancer) will decrease, whereas the proportion of samples showing “noise” (abnormal cytology results due to inflammation and reactive changes that are self-limiting) will increase. The positive predictive value of minor cytological abnormalities will be even lower because of the reduced prevalence of CIN. As a result, because of its subjective nature, the reading and interpreting cytology results will be even more prone to human error. For these reasons, assays based on objective methods such as the detection of HPV and biomarkers will be advantageous.

Since HPV is a sexually-transmitted infection, using HPV testing for cervical screening may lead to anxiety and concerns about sexual relationships, and may have an emotional impact on the quality of life and a negative impact on mental health^272^.

Other practical challenges are the diversion of resources towards vaccination, and the resulting pressure for less frequent screening. At present, the recommended frequency in most countries is not less than once every 2–3 years. To lengthen the screening interval means getting closer to the interval required for advancing from low-grade to high-grade intraepithelial lesions or even to the development of invasive cancer. In other words, there may be just one chance to pick up at-risk women before the full development of invasive cancer. This will require a test with extreme sensitivity that is subject to a low positive predictive value, especially in the vaccinated population with a low incidence of disease. Lengthening of the interval for cytological examination is not advisable because the sensitivity of the test is low and repeated negative cytological tests are required to ensure no underlying precancerous or cancerous lesions. The high sensitivity of HPV testing, on the other hand, is more reassuring and allows lengthening of the screening interval. To overcome the low positive predictive value of highly-sensitive HPV testing in the vaccinated population with a low incidence of disease, a supplementary test for other biomarkers or co-testing with cytology is the way to move forward.

## Conclusions

Technically speaking, HPV testing has several advantages over cytology-based screening, particularly in the situation where the incidence of cervical cancer and precancer decreases substantially following the widespread use of vaccination. However, although it is beyond the scope of this review, the psycho-social stigma associated with testing for a sexually-transmitted infection needs to be addressed. It has been shown that, even among well-educated women, most have not heard of HPV and do not know its association with cervical cancer^273^–^276^. Public education should go in parallel with the technical development in HPV testing. Moreover, whatever technology is used for screening, it is important to point out that the key for success is to reach a high coverage. Screening for cancer is like looking for a needle in a haystack, which is always challenging.

## Abbreviations

ASCUS: atypical squamous cells of undetermined signifcance
ASC-H: atypical squamous cells – cannot exclude high-grade squamous intraepithelial lesion
CIN: cervical intraepithelial neoplasia
EV: epidermodysplasia verruciformis
HC2: hybrid capture assay version 2
HPV: human papillomavirus
HSIL: high-grade squamous intraepithelial lesion
IS: international standard
LEEP: loop electrosurgical excision procedure
LSIL: low-grade squamous intraepithelial lesion
LCR: long control region
MCMP: minichromosome maintenance proteins
ORF: open reading frame
PCR: polymerase chain reaction
Rb: retinoblastoma
TOP2: topoisomerase II
US FDA: United States Food and Drug Administration
WHO: World Health Organization
